# HSTLBO: A hybrid algorithm based on Harmony Search and Teaching-Learning-Based Optimization for complex high-dimensional optimization problems

**DOI:** 10.1371/journal.pone.0175114

**Published:** 2017-04-12

**Authors:** Shouheng Tuo, Longquan Yong, Fang’an Deng, Yanhai Li, Yong Lin, Qiuju Lu

**Affiliations:** School of Mathematics and Computer Science, Shaanxi University of Technology, Hanzhong, P.R. China; Beihang University, CHINA

## Abstract

Harmony Search (HS) and Teaching-Learning-Based Optimization (TLBO) as new swarm intelligent optimization algorithms have received much attention in recent years. Both of them have shown outstanding performance for solving NP-Hard optimization problems. However, they also suffer dramatic performance degradation for some complex high-dimensional optimization problems. Through a lot of experiments, we find that the HS and TLBO have strong complementarity each other. The HS has strong global exploration power but low convergence speed. Reversely, the TLBO has much fast convergence speed but it is easily trapped into local search. In this work, we propose a hybrid search algorithm named HSTLBO that merges the two algorithms together for synergistically solving complex optimization problems using a self-adaptive selection strategy. In the HSTLBO, both HS and TLBO are modified with the aim of balancing the global exploration and exploitation abilities, where the HS aims mainly to explore the unknown regions and the TLBO aims to rapidly exploit high-precision solutions in the known regions. Our experimental results demonstrate better performance and faster speed than five state-of-the-art HS variants and show better exploration power than five good TLBO variants with similar run time, which illustrates that our method is promising in solving complex high-dimensional optimization problems. The experiment on portfolio optimization problems also demonstrate that the HSTLBO is effective in solving complex read-world application.

## 1 Introduction

With the scientific and social progress, new complex problems are more and more encountered in the fields of science and engineering. Especially, many high-dimensional optimization problems in engineering design, production scheduling and scientific calculation need urgently to be solved with high performance and high efficiency, for which there are three challenges: the first one is the very large search space owing to the very high dimensional problems (e.g., >500), which makes the enormous computation burden; the second one is the large number of modals (multi-extremum points), which makes the search algorithm be easily trapped into local search; the third one is the particularity of optimization problem that may be discontinuous, non-differentiable and even have no objective function, for which the traditional mathematical optimization algorithms are powerless due to the requiring of substantial gradient information. Therefore, it is of great challenge to discover the globally optimal solution in an efficient time for solving a complex multimodal optimization problem with more than 1000 dimensions, possibly infinite number of local minima and non-differentiable.

To address complex optimization problems, the swarm intelligent algorithms, mimicking the collective behavior of decentralized, self-organized systems, natural or artificial, have received much attention in recent years, most of which are nature inspired, such as genetic algorithm (GA)[1] inspired by biological evolution, particle swarm optimization (PSO)[2] mimicking the foraging process of bird flock, differential evolution (DE)[3], artificial bee colony (ABC)[4], Symbiotic organisms Search (SOS)[5] and so on. Comparing with traditional mathematical optimization algorithms, the swarm intelligent optimization algorithms are not limited by requiring substantial gradient information and not dependent on an initialization.

Both Harmony Search (HS) [6–7] and Teaching-Learning-Based Optimization (TLBO) [8–9] are new swarm intelligent optimization methods that have attracted increasing interests owing to their excellent characteristics, such as less parameters, simplicity, utilizing real-number encoding and fewer mathematical requirements and so forth. The advantage of HS is that it maintains population diversity very well during the search process; it has strong exploration power for exploring the unknown space. TLBO is powerful in obtaining extraordinary precision solution due to having very strong convergence ability. However, the HS and TLBO algorithms also have some limitations for solving high-dimensional optimization problems with multimodality. The HS has disadvantages on convergence speed, precision of globally optimal solution over TLBO; conversely the TLBO is easy to fall into a local search owing to convergence speed very rapid, which easily results in globally optimal solution lost for some multimodal problems.

In the case, several HS variants [10–25] and modified TLBO algorithms [26–29] have been presented to improve the performance for solving complex optimization problems in recent years, such as, SGHS[11], IHS[12], ITHS[14], EHS[15], NGHS[18], DIHS[19], NDHS[20], DSHS[23], ATLBO [26], WTLBO[27], TLBO_GC[28], ITLBO[29] and so on [30–31]. However, these improved variants still are not competent enough to tackle the optimization problems with high-dimensionality (larger than 500) and multimodality. For example, the solution precision of IHS is not satisfactory; NGHS, SGHS and NDHS are easy to trap into local search. EHS and DSHS require taking much time for high-dimensional problems; WTLBO, TLBO_GC and ITLBO cannot avoid premature convergence for complex optimization problems with multimodality. The reason is these state-of-the-art intelligent algorithms have not considered an important that, with the increase of dimensionality, the probability that all values of one of dimensions in population became assimilated and lost the diversity will increase, which will make the algorithm lose exploration power if the algorithm has not good disturbance strategy for escaping from the local search. In this work, we find from the merit and demerit of HS and TLBO that the HS and TLBO have many complementary performance each other. We think it is a viable way in solving the high-dimensional optimization problems with multimodality if a good integration of HS and TLBO can be realized.

Commonly, to solve multimodal optimization problems that have one globally optimal solution and many local minimum (maximum) values by employing swarm intelligent algorithm, the overarching goal is to effectively implement the balance between exploration power and exploitation power, where the mission of exploration is to discover the unexplored regions at the early stage of search process, and the exploitation aims to obtain high precision optimal solution in a known region that has been found in the exploration stage. As a consequence, balancing the exploration and the exploitation is very important for solving a high-dimensional optimization with multimodality. Generally, the exploration power is strongly required before discovering the region in which the globally optimal solution is contained inside, however, after the globally optimal region has been found, the exploitation power should be intensified immediately and the exploration power is degraded gradually.

To address the balance, in this study, we propose a hybrid optimization algorithm (HSTLBO) based on HS and TLBO, in which a self-adaptive selection strategy is designed to balance the exploration power and the exploitation power. At the early stage of search, the HS algorithm obtains higher probability for exploring the region that contains the globally optimal solution. When the globally optimal region might have been located, the TLBO algorithm begins to obtain higher probability at the later stage of search process for intensifying the local search and exploiting high precision solution. In the HSTLBO, a self-adaptive selection probability is used to choose HS or TLBO in terms of the population diversity and update-success-rate. The update-success-rate denotes the proportion that the new generated solutions are superior to the old ones, which mean the rate that a new generated solution can successfully replace the old one in one generation.

The rest of this paper is organized as follows: Section 2 introduces the HS and TLBO algorithm. Hybrid HSTLBO algorithm is proposed and self-adaption selection strategy is analyzed in Section 3. In Section 4, the numerical experiments on twenty complex benchmark test functions are performed, the results are analyzed by comparing and statistical test. And the convergence of HSTLBO is also investigated. In Section 5, HSTLBO is used to solve portfolio optimization problem. Section 6 concludes this work.

Some symbols are explained as Box 1.

**Box 1**

NP  --The population size which is equal to HMS.

T_max_ --The maximum evaluation times of objective function.

t   --The current iteration times.

SR  --the selection rate that new generated harmony insteads of the worst harmony.

T   --The cycle length for recalculating the SR.

r   --An uniform distributed random number between 0 and 1.

c1/ c2 --The times of updating old solutions successfully of HS/ TLBO in the t^th^ iteration.

## 2 Harmony Search and Teaching-Learning-Based Optimization

### 2.1 Optimization model

$$\underset{X}{\text{min}}f(X),X=\left(x_{1},x_{2},\ldots ,x_{D}\right)$$

$$\text{S}.\text{t}.\;x_{i}\in \left[x_{i}^{L},x_{i}^{U}\right],i=1,2,\ldots ,D$$

Where, *X* consists of *D* decision variables (*x*_1,_ *x*_2,_ …, *x*_*D*_), *D* denotes the dimensionality (the number of decision variables) of the optimization problem, *x*_*i*_ (*i* = 1, 2, …, *D*) represents the i^th^ decision variable. $x_{i}^{U}$ and $x_{i}^{L}$ separately indicate the upper and lower bound of *x*_*i*_.

### 2.2 HS algorithm

**Fig 1:**
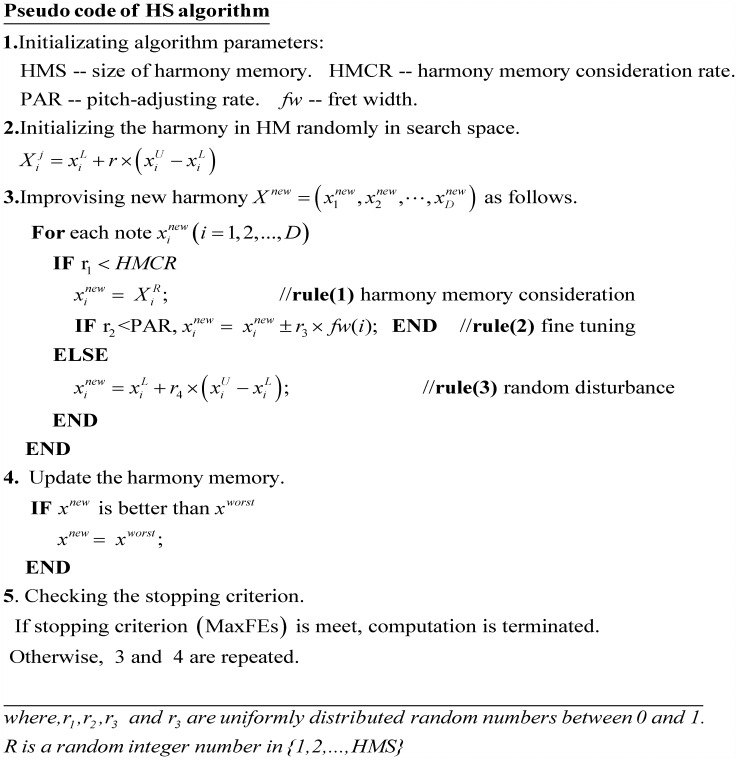
Pseudo code of standard HS algorithm.

**HS algorithm** mimics the process of improvising a musical harmony, in which *X* denotes the harmony, *x*_*i*_(i = 1, 2,…, D) indicates the note of harmony, *D* is the number of notes in a harmony. Harmony memory (HM) contains HMS harmonies: {*X*^1^, *X*^2^, …, *X*^*HMS*^}, where $X^{j}=\left(x_{1}^{j},x_{2}^{j},\ldots ,x_{D}^{j}\right)$, HMS denotes the population size. The pseudo code of HS algorithm is as Fig 1.

In standard HS, three operators (harmony memory consideration, pitch-adjusting and random disturbance) are employed to optimize the harmonies in HM (population), which are good at exploring new region of search space [14]. However, due to the absence of learning-operator, the convergence speed of HS is much slower than that of TLBO.

### 2.3 TLBO algorithm

A new swarm intelligent optimization algorithm, TLBO, is proposed by R.V.Rao in 2012, which is inspired by the teaching and learning process of a class [8]. In TLBO, The class {*X*^1^, *X*^2^,…, *X*^*NP*^} is composed of one teacher and some learners, where $X^{j}=\left(x_{1}^{j},x_{2}^{j},\ldots ,x_{D}^{j}\right)$ (*j* = 1, 2,…, NP) (see Box 1) denotes the j^th^ learner, NP is the class size, *D* represents the number of major subjects in the class; the $x_{i}^{j}$ represents the learning status of j^th^ learner on i^th^ major subject. The optimization process of TLBO is divided into two stages: “teacher phase” and “learner phase”.

#### Teacher phase

In the teacher phase, the learners increase their knowledge depending on the teacher who tries to improve the mean ability of all learners. The teaching operator is as follow,

$$\begin{array}{l} X^{j,new}=X^{j,old}+r\times (X^{teacher}-T_{F}\times M)\\ M=\frac{1}{NP}\sum_{j=1}^{NP}X^{j}\\ T_{F}=round\left(1+rand(1,D)\right) \end{array}$$

Where *X*^*j*,*new*^ and *X*^*j*,*old*^ denote the i^th^ learner’s learning status after and before learning from teacher *X*^*teacher*^, *T*_*F*_ is teaching factor, *r* is a uniformly distributed random vector in the range [*x*^*L*^, *x*^*U*^]. *M* denotes the mean knowledge level of all learners.

#### Learner phase

In the learner phase, each learner increases knowledge depending on communicating with other learners. The learning operator is as follow,

$$X^{j,new}=\{\begin{array}{c} X^{j,old}+r\times (X^{r}-X^{j,old}),f(X^{r})<f(X^{j,old})\\ X^{j,old}+r\times (X^{j,old}-X^{r}),\;otherwise \end{array}$$

Where *r*(*r* ≠ *j*) is a random integer in the range **[**1, NP**]**.

## 3. Proposed HSTLBO algorithm

To balance the exploration power and the exploitation power during the searching process, we propose a complementary HSTLBO algorithm.

### 3.1 HSTLBO algorithm

**Fig 2:**
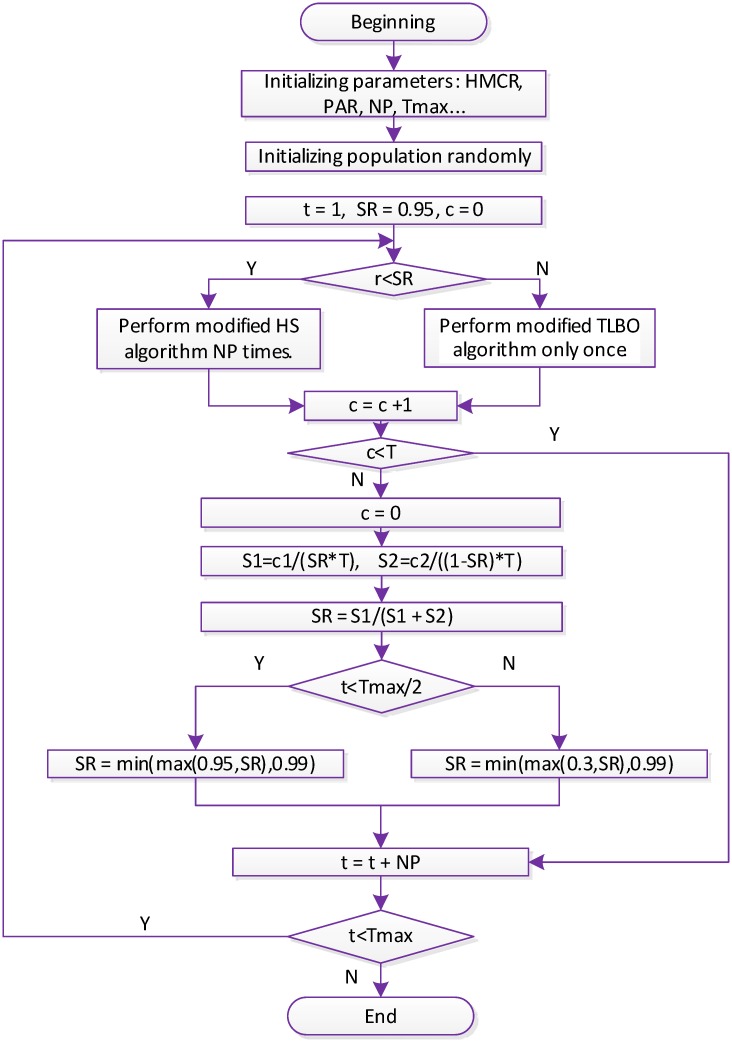
Flow chart of HSTLBO.

The flow chart of HSTLBO algorithm is as Fig 2.

In the HSTLBO algorithm, we merge HS and TLBO together to compensate for each other’s deficiencies, where the HS is mainly used for exploring the search space, and the TLBO aims to speed up the exploitation process. In the search process of HSTLBO, the HS and the TLBO compete for the opportunity in each iteration according to a self-adaptive selection rate (SR) (see Box 1). The selection rate (SR) is dynamically changed in terms of the success times that new generated solution is superior to the worst solution of population during T cycle. In the beginning stage, the HS can obtain more opportunity for exploring the unknown regions, when the region containing the globally optimal solution has been found in the later stage, the TLBO will obtain more opportunity for exploiting high-precision solution.

For a new unknown problem, exploring the unknown space in the beginning stage is the first consideration for the HSTLBO algorithm, for which the HS is a good choice. As a consequence, a high selection rate SR (≥ 0.95) is assigned to HS algorithm for exploring unknown regions in the first half of search. In the remaining time, the value of SR will continue to adapt the population status. If the global region has not been found before or the space distribution of population is still extensive, the HS might be still able to obtain a high SR for exploring the unknown areas. However, if the HS obtains no or very low selection rate, (in other words, the TLBO has very high probability), the diversity of population might be lost quickly for high-dimensional optimization problems owing to the quick convergence of TLBO. Therefore, to keep the diversity of population in a certain level, in the second half of stage, the HS is also given more than 0.3 probabilities to run.

In the hybrid HSTLBO algorithm, both HS and TLBO are modified as follows.

### 3.2 Modified HS

**Fig 3:**
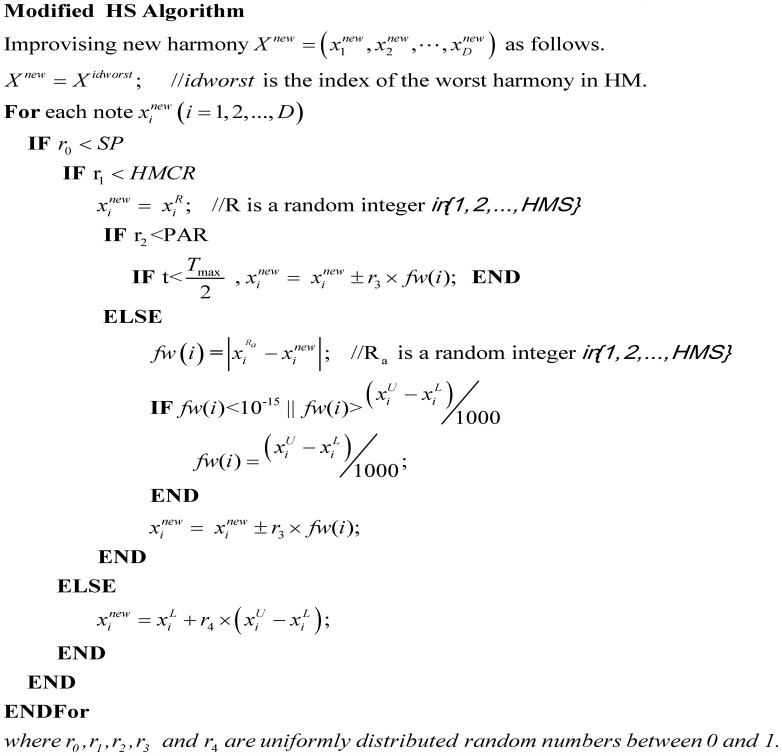
Pseudo code of modified HS algorithm.

In the modified HS algorithm (see Fig 3), all the steps are identical to the steps of standard HS algorithm except for step 3 (improvising a new harmony). The key difference of step 3 between modified HS and standard HS are:

1. Standard HS algorithm produces a new harmony in which each element (decision variable) is generated based on three HS rules (a. harmony memory consideration; b. pitch-adjusting; c. randomly disturbance). In our proposed algorithm, the producing process of new harmony is similar to that of DIHS [19]. Dynamic selection strategy is adopted to select some elements of the worst harmony with probability *SP* for adjusting. The formula of *SP* is expressed in Eq (1).
  $$SP=\text{min}\left[\frac{100}{D}-\left(\frac{100}{D}-\frac{30}{D}\right)\times {\left(\frac{t}{T_{\text{max}}}\right)}^{2},1\right]$$ (1)
  The selection probability *SP* is adjusted dynamically according to current iteration *t*, which is introduced in DIHS [19].
2. In the standard HS, parameters *PAR* and *fw* are constant values. The modified HSTLBO algorithm adopts dynamic strategies to change *PAR* and *fw* for balancing the exploration power and the exploitation power (see Eqs (2) and (3)). The *fw* is divided into two stages. In the first half of the generation, the *fw* is dynamically changed with the increasing of iteration, which is the same as the *bw* in IHS [12]. The overarching goal of *fw* is to maintain strong exploration power. In the second half of generations, in order to adapt the characteristics of problems, the value of *fw* is changed adaptively in terms of the values of individuals.
  $$PAR=PAR_{\text{min}}+\left(PAR_{\text{max}}-PAR_{\text{min}}\right)\times \frac{t}{T_{\text{max}}}$$ (2)
  $$\begin{array}{l} fw\text{(}i)=\left\{ \begin{array}{l} fw_{\text{max}}(i)\times \text{exp}\left({\left(\frac{t}{T_{\text{max}}}\right)}^{2}\times \text{log}\frac{fw_{\text{min}}(i)}{fw_{\text{max}}(i)}\right),t<T_{\text{max}}\\ \left|x_{i}^{{}^{R_{a}}}-x_{i}^{new}\right|,t\geq T_{\text{max}} \end{array}\right.,i=1,2,\ldots ,D \end{array}$$ (3)

### 3.3 Modified TLBO

**Fig 4:**
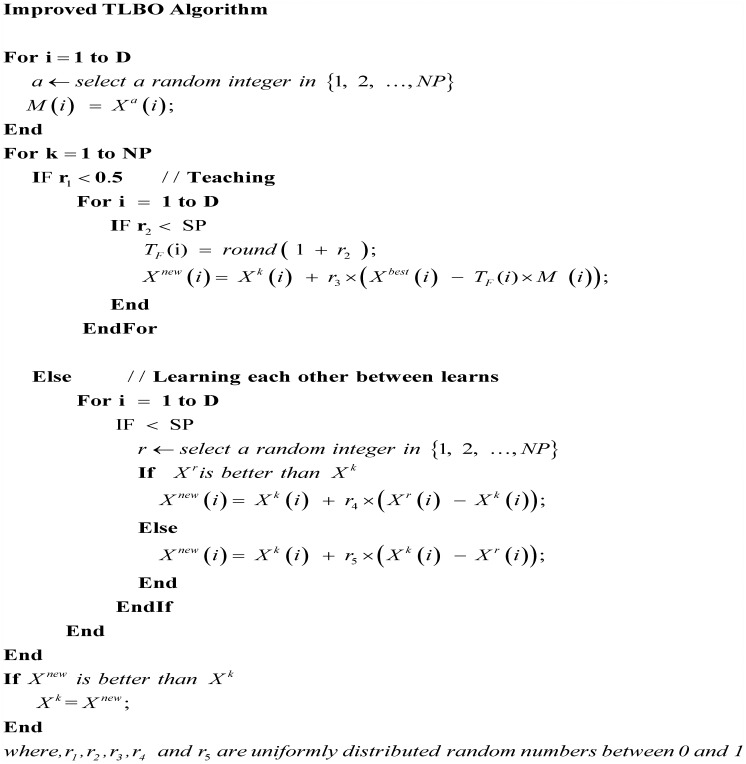
Pseudo code of modified TLBO algorithm.

The primary difference between standard TLBO and modified TLBO **(see** Fig 4**)** is as follows.

1. In standard TLBO, *M* is equal to the mean value of learners *X*. In modified TLBO, it is a combination vector in which each subject *M*(*i*) is randomly chosen from the i^th^ subject of all learners.
2. In each iteration. TLBO performs teacher phase and learner phase, respectively. The modified TLBO only randomly chooses either teacher phase or learner phase to perform.
3. In standard TLBO, all dimensions of *X*^*new*^ are produced by learning from teacher or other one learner. Whereas, in modified TLBO, only a portion of dimensions of *X*^*new*^ are generated by learning from teacher or other one learner, and other dimensions inherit from *X*^*old*^ directly, which is because an excellent learner is also imperfect on some subjects, selective learning on parts of subjects is more effective for improving knowledge level of learner than learning all subjects from one learner. The selection probability SP is shown in Eq (1), the selection of parameters has been explained and analyzed in our proposed DIHS algorithm [19].
4. As we known, in our real lives, selective learning from multiple excellent learners on some subjects is more effective for improving our knowledge level than learning all subjects only from one excellent learner. As a consequence, in the modified TLBO, the learner on each subject will select one other learner from population for better learning new knowledge.

## 4. Experimental study

To investigate the performance of proposed HSTLBO algorithm, numerical simulation experiments on twenty benchmark functions [32–35] are tested. Parameter settings are listed for all compared HS and TLBO variants in Table 1.

**Table 1:** Algorithm parameter settings.

Algorithm	Population size	HMCR	PAR	*fw* (*bw*)
HS	10	0.99	0.33	0.01
IHS	10	0.90	PAR_max_ = 0.99; PAR_max_ = 0.1	*fw*_*max*_ *=* (x^U^-x^L^)/1000; *fw*_*min*_ *=* 0.0001
ITHS	10	0.99	PAR_max_ = 1; PAR_max_ = 0	/
NDHS	10	0.99	PAR_max_ = 0.99; PAR_max_ = 0.1	/
DSHS	10	0.99	PAR_max_ = 0.99; PAR_max_ = 0.1	/
EHS	10	0.99	0.33	/
HSTLBO	10	0.99	PAR_max_ = 0.9; PAR_max_ = 0.1	*fw*_*max*_ *=* (x^U^-x^L^)/1000; *fw*_*min*_ *=* 1e-10
**Algorithm**	**Population size**			
TLBO	10	/		
ATLBO	10	/		
WTLBO	10	*w*_min_ = 0.1,*w*_max_ = 0.9		
TLBO-GC	10	/		
ITLBO	10	Pc = 0.8; M = 5; (rearrange) m = 100		

**Twenty** well-known functions are listed in Table 2, which include 16 multimodal functions (F_1_-F_6_, F_8_, F_12_-F_20_) and 4 complex uni-modal problems (F_7_, F_9_-F_11_), 4 hybrid functions (F_17_-F_20_) and 10 shift functions (F_9_-F_18_).

**Table 2:** Sixteen complex benchmark functions (F_1_-F_20_).

Function Name	Search Range	Optimum Value	Function Type Multi/Shifted/Separable/Hybrid
F_1_:Ackley Function	**[**–32,32**]** ^D^	X* = (0,0,⋯,0), *F*(X*) = 0	Y/N/Y/N
F_2_: Griewank Function	**[**–600,600**]** ^D^	X* = (0,0,⋯,0), *F*(X*) = 0	Y/N/N/N
F_3_:Levy Function	**[**–10,10**]** ^D^	X* = (1,1,⋯,1), *F*(X*) = 0	Y/N/N/N
F_4_:Michalewics Function	**[**-10, π**]** ^D^	unknown	Y/N/N/N
F_5_:Rastrigin Function	**[**-5.12,5.12**]**^D^	X* = (0,0,⋯,0), *F*(X*) = 0	Y/N/N/N
F_6_:Schwefel 2.26 Function	**[**–512,512**]**^D^	X* = (420.9687,420.9687,⋯,420.9687), *F*(X*) = 0	Y/N/N/N
F_7_:Rosenbrock Function	**[**–100,100**]**^D^	X* = (1,1,⋯,1), *F*(X*) = 0	N/N/N/N
F_8_:Schwefel2.22 Function	**[**–5,5**]**^D^	X* = (0,0,⋯,0), *F*(X*) = 0	Y/N/N/N
F_9_:Sphere Shift Function	**[**–100,100**]**^D^	X* = *o*, *F*(X*) = 0	N/Y/N/N
F_10_:Schwefel_Shift Function	**[**–100,100**]**^D^	X* = *o*, *F*(X*) = 0	N/Y/N/N
F_11_:Rosenbrock shift Function	**[**–100,100**]**^D^	X* = *o*, *F*(X*) = 0	N/Y/N/N
F_12_:Griewank Shift Function	**[**–600,600**]**^D^	X* = *o*, *F*(X*) = 0	Y/Y/N/N
F_13_:Rastrigin Shift Function	**[**-5.12,5.12**]**^D^	X* = *o*, *F*(X*) = 0	Y/Y/N/N
F_14_:Ackley Shift Function	**[**–32,32**]**^D^	X* = *o*, *F*(X*) = 0	Y/Y/N/N
F_15_:FastFractal ‘DoubleDip’ unction	**[**–1,1**]**^D^	Unknown	Y/Y/N/N
F_16_: Schaffer Shift function	**[**–100,100**]**^D^	X* = *o*, *F*(X*) = 0	Y/Y/N/N
F_17_: Extended_f10 Shift Function	**[**–100,100**]**^D^	x* = *o*, F_21_(x*) = 0	Y/Y/N/Y
F_18_: Bohachevsky Shift Function	**[**–15,15**]**^D^	x* = *o*, F_22_(x*) = 0	Y/Y/N/Y
F_19_: Extended Function	**[**–100,100**]**^D^	x* = *o*, F_21_(x*) = 0	Y/N/N/Y
F_20_: Bohachevsky Function	**[**–15,15**]**^D^	x* = *o*, F_22_(x*) = 0	Y/N/N/Y

In the simulation experiment, all the test programs were performed on Windows XP 32 system with Intel(R) Core(TM) i3-2120 CPU@3.30 GHz and 4 GB RAM, and all the program codes were written in MATLAB R2014b.

In our simulation experiments, the dimension of test functions is set to 1000 and each function is run independently 20 times with 5E+6 function evaluations (FEs) as the termination condition. The precision (=|*f*(*X*^*best*^)−*f*(X*)|) (Prec), standard deviation of the precision (Std dev) and mean run time (Mtime) for each function are calculated over 20 independent runs, where *X*^*best*^ is the best solution in population when the terminate condition is meet, and X* is the global optimal solution.

### 4.1. Comparison with state-of-the-art HS variants

**Fig 5:**
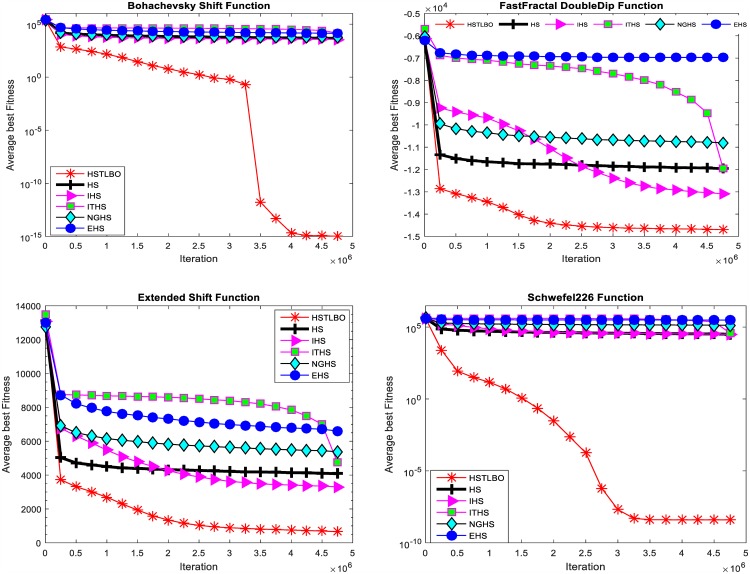
Convergence curves of four functions.

**Fig 6:**
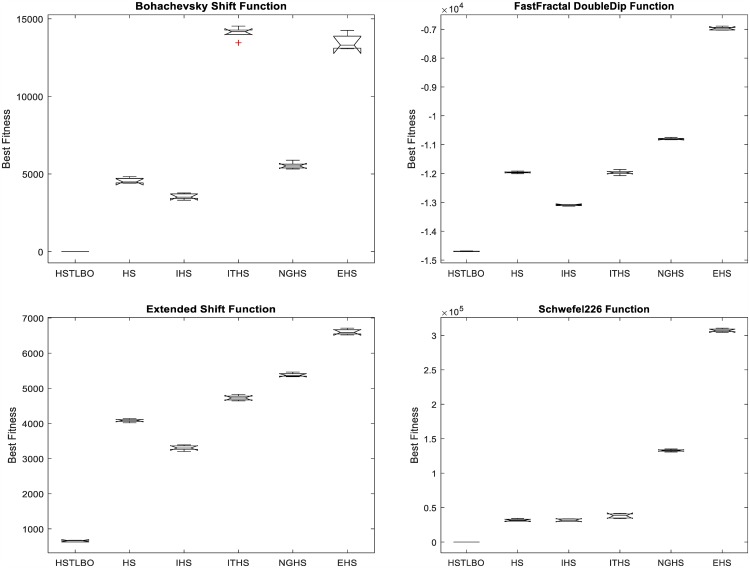
Box plots of four functions.

HSTLBO is compared with standard HS algorithm and four state-of-the-art HS variants: IHS [12], ITHS [14], EHS [15], and NGHS [18]. To ensure the comparison fair, for all compared algorithms, each test function is run 20 times independently with 5E+6 FEs as the terminal criterion. The parameters of five HS variants are set the same value as the recommended value of original paper. The experimental results of six compared algorithms are summarized in Table 3. Fig 5 displays the convergence curve and box plots of distribution of optimal solutions after 20 independent runs are displayed in Fig 6.

1. **Quality of solution**. For twenty test functions with dimension of 1000, the results provided by the five HS variants are far away from the global optimal solution. However, it can be found from Table 3 that HSTLBO is obviously superior to other algorithms, and the optimal solutions obtained by the proposed algorithm are very close to the global optimal solutions for all test functions except for F_7_,F_10_,F_11_,F_15_-F_17_.
2. **CPU runs time.** It can be seen from Table 3 that the HSTLBO algorithm takes less run time for all 20 test functions than other five HS variants.
3. **Robustness**. From Fig 5, the convergence curves of the HSTLBO is much active in whole search process, which demonstrates that the HSTLBO algorithm can maintain the strong search ability during the search process. Nevertheless, the other HS algorithms are easy convergence premature, which demonstrate the algorithms lose the search ability owing to the stagnation of the search. The boxplots (see Fig 6) indicate that optimal solutions of the HSTLBO have a more narrow distribution than those of other algorithms, which illustrates that our method has strong stability and robustness on 20 runs.

**Table 3:** Experimental results of HS, IHS, ITHS, NGHS, EHS and HSTLBO over 20 independent runs on 20 test functions of 1000 variables with 5E+6 FEs. “Prec” and “Std Dev” denote the precision and standard deviation of the function error values in 20 runs, respectively. Time(s) is the mean run time over 20 independent runs on 5000000 FEs.

	ALG	Prec	Std Dev	Mtime(s)	Prec	Std Dev	Mtime(s)	Prec	Std Dev	Mtime(s)	Prec	Std Dev	Mtime(s)
**F1~F4**	**HSTLBO**	1.34E-13	4.05E-15	3.48E+02	9.33E-16	6.08E-17	3.94E+02	2.18E-26	1.53E-27	9.20E+02	-9.93E+02	3.96E-01	1.18E+03
	**HS**	9.34E+00	1.44E-01	7.25E+02	5.13E+02	1.39E+01	8.68E+02	1.35E+02	6.20E+00	1.28E+03	-2.77E+02	3.55E+00	1.63E+03
	**IHS**	8.55E+00	8.71E-02	1.03E+03	3.71E+02	2.75E+01	1.19E+03	1.39E+02	4.96E+00	1.66E+03	-8.37E+02	5.79E+00	1.91E+03
	**ITHS**	1.62E+00	1.57E+00	8.58E+02	1.45E+01	2.45E+01	1.00E+03	6.96E+01	7.75E+00	1.50E+03	-8.16E+02	5.46E+00	1.81E+03
	**NGHS**	1.01E+01	8.98E-02	6.47E+02	6.29E+02	1.60E+01	7.51E+02	2.21E+02	7.21E+00	1.31E+03	-4.51E+02	3.25E+00	1.45E+03
	**EHS**	1.33E+01	1.14E-01	1.39E+03	1.64E+03	5.89E+01	1.52E+03	2.33E+03	1.01E+02	2.04E+03	-2.43E+02	1.30E+00	2.22E+03
**F5~F8**	**HSTLBO**	4.37E-12	1.63E-12	2.63E+02	3.91E-09	1.77E-10	3.29E+02	1.89E+03	8.93E+01	2.33E+02	6.09E-21	1.48E-21	2.13E+02
	**HS**	1.44E+03	7.50E+00	6.46E+02	3.18E+04	1.73E+03	7.58E+02	1.03E+05	5.88E+03	5.55E+02	2.02E+02	1.62E+00	5.88E+02
	**IHS**	1.57E+03	3.98E+01	1.03E+03	3.17E+04	1.79E+03	9.43E+02	9.35E+04	1.25E+03	9.12E+02	1.37E+02	3.53E+00	8.99E+02
	**ITHS**	6.39E+00	4.60E+00	8.85E+02	3.79E+04	3.35E+03	9.87E+02	7.21E+04	1.61E+04	8.64E+02	1.66E+01	1.16E+01	8.13E+02
	**NGHS**	4.70E+03	7.99E+01	5.76E+02	1.33E+05	1.57E+03	6.88E+02	9.72E+04	7.18E+03	4.86E+02	2.98E+02	5.58E+00	4.75E+02
	**EHS**	1.13E+04	6.36E+01	1.35E+03	3.07E+05	2.37E+03	1.22E+03	3.03E+06	1.58E+05	1.18E+03	3.89E+02	5.60E+00	1.13E+03
**F9~F12**	**HSTLBO**	5.55E-24	3.40E-25	2.08E+02	2.81E+01	5.73E-01	2.11E+02	1.32E+03	1.85E+02	2.58E+02	5.37E-15	3.73E-16	4.90E+02
	**HS**	6.77E+04	1.24E+03	5.69E+02	5.55E+01	7.77E-01	5.32E+02	2.17E+09	1.68E+08	6.02E+02	6.02E+02	2.32E+01	9.02E+02
	**IHS**	5.39E+04	1.21E+03	9.28E+02	6.05E+01	9.71E-01	8.85E+02	2.14E+09	1.45E+08	9.72E+02	4.70E+02	1.66E+01	1.26E+03
	**ITHS**	4.38E+05	1.54E+04	7.96E+02	6.52E+01	5.59E-01	8.02E+02	8.48E+10	4.63E+09	8.62E+02	3.84E+03	1.90E+02	1.16E+03
	**NGHS**	7.70E+04	4.15E+03	4.67E+02	4.40E+01	4.25E-01	4.63E+02	1.63E+09	1.61E+08	5.40E+02	6.97E+02	6.20E+00	7.50E+02
	**EHS**	1.63E+05	6.80E+03	1.16E+03	1.28E+02	1.91E+00	1.13E+03	5.22E+10	4.04E+09	1.17E+03	1.46E+03	5.44E+01	1.41E+03
**F13~F16**	**HSTLBO**	5.90E-14	1.27E-14	2.72E+02	8.11E-13	2.43E-14	3.51E+02	3.02E+02	9.86E+00	1.56E+03	6.63E+02	3.75E+01	2.13E+03
	**HS**	1.58E+03	1.94E+01	6.25E+02	9.56E+00	1.23E-01	7.27E+02	3.04E+03	3.33E+01	1.87E+03	4.00E+03	4.54E+01	2.39E+03
	**IHS**	1.75E+03	3.12E+01	8.50E+02	9.15E+00	2.67E-01	8.44E+02	1.91E+03	2.50E+01	2.25E+03	3.32E+03	8.09E+01	3.13E+03
	**ITHS**	4.53E+03	1.79E+02	1.02E+03	1.58E+01	1.48E-01	8.74E+02	3.03E+03	7.55E+01	2.10E+03	4.78E+03	1.25E+02	2.85E+03
	**NGHS**	4.93E+03	1.10E+02	6.86E+02	1.01E+01	1.26E-01	5.75E+02	4.20E+03	3.28E+01	1.92E+03	5.29E+03	7.02E+01	2.47E+03
	**EHS**	1.01E+04	6.64E+01	1.20E+03	1.31E+01	8.10E-02	1.07E+03	8.03E+03	5.64E+01	2.77E+03	6.65E+03	7.44E+01	3.45E+03
**F17~F20**	**HSTLBO**	6.53E+02	2.48E+01	2.37E+03	1.02E-15	1.99E-16	7.10E+02	3.94E-10	6.58E-11	2.04E+03	0.00E+00	0.00E+00	3.86E+02
	**HS**	4.08E+03	4.39E+01	2.56E+03	4.57E+03	1.80E+02	1.22E+03	4.06E+03	1.16E+02	2.40E+03	4.64E+03	1.22E+02	9.94E+02
	**IHS**	3.31E+03	7.20E+01	2.87E+03	3.56E+03	1.84E+02	1.59E+03	3.33E+03	5.52E+01	2.72E+03	3.46E+03	1.08E+02	1.31E+03
	**ITHS**	4.73E+03	7.23E+01	2.73E+03	1.41E+04	3.94E+02	1.32E+03	3.85E+02	2.09E+02	2.48E+03	7.33E+02	5.46E+02	1.09E+03
	**NGHS**	5.38E+03	5.13E+01	2.69E+03	5.54E+03	2.14E+02	1.02E+03	5.33E+03	1.45E+02	2.43E+03	5.28E+03	2.05E+02	7.25E+02
	**EHS**	6.61E+03	7.71E+01	3.45E+03	1.35E+04	5.02E+02	1.81E+03	6.68E+03	6.27E+01	3.21E+03	1.26E+04	1.94E+02	1.46E+03

### 4.2 Comparison with TLBO variants

In this section, we compare the HSTLBO with standard TLBO and four state-of-the-art TLBO variants: ATLBO [26], WTLBO [27], TLBO_GC [28], and ITLBO [29]. In ATLBO, elitist strategy, weight function and acceleration coefficient are employed to improve the performance. WTLBO introduces a weighted TLBO algorithm for balancing the exploration and the exploitation. In TLBO_GC, a global crossover strategy is proposed for solving global optimization problems. ITLBO adopts a local learning and self-learning methods for improving the global search ability of TLBO.

**Fig 7:**
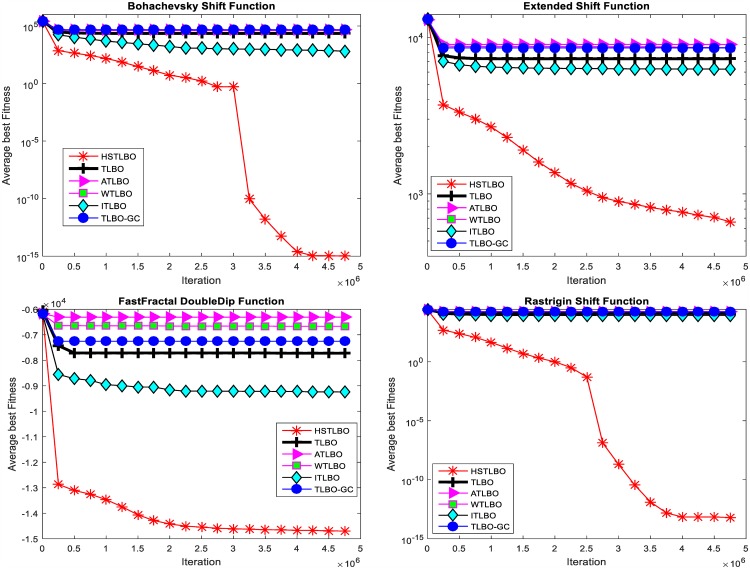
Convergence curves of four functions.

**Fig 8:**
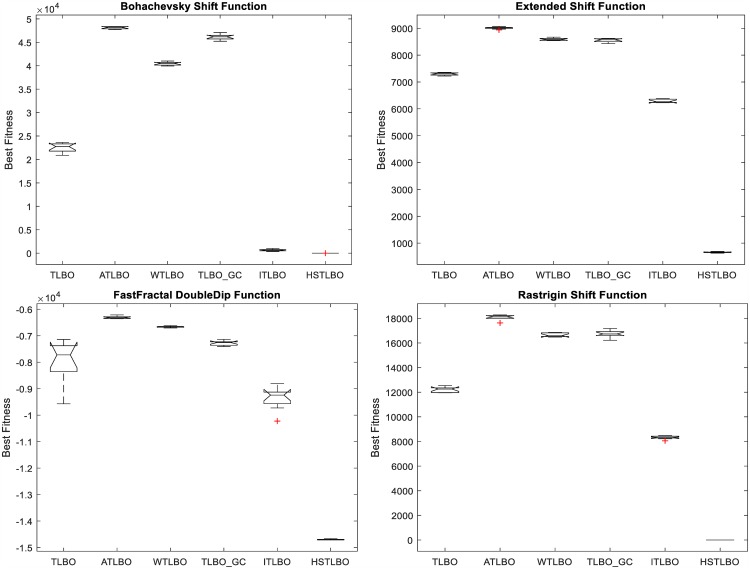
Box plots of four functions.

To ensure the comparison fair, for all compared TLBO algorithms, each test function is run 20 times independently with 5E+6 FEs as the terminal criterion. The experimental results of six compared algorithms are summarized in Table 4. Fig 7 displays the convergence curves and Fig 8 shows the distribution of optimal solutions on 20 independent runs for six TLBO algorithms using box plots.

1. Quality of solution. In twenty test functions, our method is the winner on 13 functions and is very close to the winner algorithm on other seven functions. Especially, for the complex **multimodal** functions, such as F_3_, F_6_, F_9_, F_13_-F_15_ and F_18_, the precision of proposed algorithm is much better than other five TLBO variants. For functions F_1_, F_2_, F_5_, F_7_, F_8_ and F_19_, the optimal solution of our method is worse than those of other algorithms, however, the solutions of our method are on the verge of global optimal solutions, which are acceptable to the application.
2. Robustness. It can be seen from Fig 7 that, comparing with five TLBO algorithms, the convergence curves of the HSTLBO is also much more active in the search process than those of other TLBO algorithms, and the HSTLBO has strong exploitation power (the convergence curve keeps decreasing) in the later stage of search process, which demonstrates that the HSTLBO can keep diverse distribution of population during the search process. From the boxplots (see Fig 8), we can find the HSTLBO is more stable on obtaining the optimal solution.

**Table 4:** Experimental results of TLBO, ATLBO, WTLBO, TLBO_GC, ITLBO and HSTLBO over 20 independent runs on 20 test functions of 1000 variables with 5E+6 FEs. “Prec” and “Std Dev” denote the precision and standard deviation of the function error values in 20 runs, respectively. Time(s) is the mean run time over 20 independent runs on 5000000 FEs.

	ALG	Prec	Std Dev	Mtime(s)	Prec	Std Dev	Mtime(s)	Prec	Std Dev	Mtime(s)	Prec	Std Dev	Mtime(s)
**Prec**	**Std Dev**	**Mtime(s)**	1.83E-15	1.62E+02	0.00E+00	0.00E+00	2.88E+02	8.53E+01	1.42E+00	9.07E+02	5.70E+02	1.48E+02	1.17E+03
	**ATLBO**	4.44E-15	0.00E+00	1.50E+02	0.00E+00	0.00E+00	2.97E+02	9.14E+01	1.19E-02	8.95E+02	8.43E+02	1.36E+01	1.12E+03
	**WTLBO**	8.88E-16	1.50E-15	2.63E+02	0.00E+00	0.00E+00	3.31E+02	8.92E+01	2.69E-01	8.88E+02	8.49E+02	5.72E+00	1.01E+03
	**TLBO_GC**	7.99E-15	1.12E-15	5.51E+02	0.00E+00	0.00E+00	7.09E+02	9.02E+01	2.89E-01	1.25E+03	7.47E+02	6.53E+00	1.53E+03
	**ITLBO**	7.99E-15	0.00E+00	2.79E+02	1.11E-16	4.68E-17	4.28E+02	7.59E+01	2.92E+00	9.67E+02	6.04E+02	9.87E+01	1.28E+03
	**HSTLBO**	1.36E-13	4.57E-15	2.52E+02	9.99E-16	7.02E-17	4.53E+02	2.16E-26	1.10E-27	9.81E+02	6.35E+00	3.12E-01	1.19E+03
**F5~F8**	**TLBO**	0.00E+00	0.00E+00	2.06E+02	2.36E+05	6.06E+04	2.22E+02	9.93E+02	3.51E-01	1.80E+02	0.00E+00	0.00E+00	1.49E+02
	**ATLBO**	0.00E+00	0.00E+00	1.80E+02	1.43E+05	1.13E+05	1.90E+02	9.97E+02	1.97E+00	1.46E+02	0.00E+00	0.00E+00	1.21E+02
	**WTLBO**	0.00E+00	0.00E+00	2.11E+02	3.75E+05	8.43E+03	2.47E+02	9.97E+02	2.51E-01	1.75E+02	0.00E+00	0.00E+00	2.46E+02
	**TLBO_GC**	0.00E+00	0.00E+00	5.25E+02	3.66E+05	6.10E+03	6.45E+02	9.99E+02	1.06E-01	5.23E+02	0.00E+00	0.00E+00	4.61E+02
	**ITLBO**	0.00E+00	0.00E+00	2.95E+02	2.32E+05	1.45E+04	3.47E+02	9.69E+02	2.71E+00	2.21E+02	0.00E+00	0.00E+00	2.17E+02
	**HSTLBO**	3.64E-12	1.23E-12	2.94E+02	3.70E-09	1.76E-10	3.08E+02	1.94E+03	1.27E+02	2.41E+02	5.73E-21	8.83E-22	2.18E+02
**F9~F12**	**TLBO**	1.38E+06	5.25E+04	1.40E+02	9.96E+01	1.05E-01	1.47E+02	4.70E+11	2.84E+10	2.20E+02	1.25E+04	7.99E+02	4.88E+02
	**ATLBO**	3.38E+06	1.39E+04	1.17E+02	9.98E+01	6.15E-02	1.20E+02	1.28E+12	5.11E+09	2.00E+02	2.99E+04	3.14E+01	4.77E+02
	**WTLBO**	2.64E+06	4.16E+04	1.46E+02	9.83E+01	2.20E-01	1.43E+02	8.43E+11	3.98E+10	2.28E+02	2.33E+04	5.80E+02	4.91E+02
	**TLBO_GC**	3.22E+06	4.21E+04	4.92E+02	9.96E+01	1.10E-01	4.55E+02	1.18E+12	2.09E+10	5.92E+02	2.85E+04	3.48E+02	7.93E+02
	**ITLBO**	1.07E+04	6.91E+03	2.16E+02	9.91E+01	3.10E-01	2.32E+02	3.17E+06	3.35E+07	2.55E+02	1.21E+02	6.68E+01	5.31E+02
	**HSTLBO**	5.28E-24	4.72E-25	1.98E+02	2.84E+01	5.99E-01	2.18E+02	1.41E+03	1.13E+02	2.74E+02	5.33E-15	5.73E-16	4.59E+02
**F13~F16**	**TLBO**	1.23E+04	1.99E+02	2.83E+02	2.02E+01	6.65E-02	2.96E+02	7.28E+03	7.54E+02	1.48E+03	7.36E+03	3.94E+02	1.92E+03
	**ATLBO**	1.81E+04	1.86E+02	2.33E+02	2.11E+01	1.33E-02	2.45E+02	8.69E+03	4.70E+01	1.39E+03	8.99E+03	3.27E+01	1.90E+03
	**WTLBO**	1.66E+04	1.51E+02	2.92E+02	2.10E+01	4.18E-02	3.26E+02	8.33E+03	2.89E+01	1.44E+03	8.72E+03	4.01E+01	1.94E+03
	**TLBO_GC**	1.67E+04	2.61E+02	6.83E+02	2.09E+01	3.21E-02	7.45E+02	7.75E+03	9.05E+01	1.85E+03	8.66E+03	8.00E+01	2.25E+03
	**ITLBO**	8.31E+03	1.21E+02	3.56E+02	1.94E+01	1.01E-02	4.18E+02	5.76E+03	4.16E+02	1.64E+03	6.75E+03	2.02E+02	1.97E+03
	**HSTLBO**	6.31E-14	9.17E-15	3.47E+02	7.78E-13	2.62E-14	3.45E+02	2.98E+02	1.71E+01	1.61E+03	5.50E+03	2.07E+01	1.97E+03
**F17~F20**	**TLBO**	7.30E+03	4.75E+01	2.28E+03	2.27E+04	9.10E+02	7.06E+02	0.00E+00	0.00E+00	7.21E+02	0.00E+00	0.00E+00	2.15E+02
	**ATLBO**	9.01E+03	3.79E+01	2.50E+03	4.82E+04	2.51E+02	7.13E+02	0.00E+00	0.00E+00	9.91E+02	0.00E+00	0.00E+00	2.01E+02
	**WTLBO**	8.58E+03	4.39E+01	2.53E+03	4.05E+04	3.56E+02	7.51E+02	0.00E+00	0.00E+00	1.02E+03	0.00E+00	0.00E+00	2.40E+02
	**TLBO_GC**	8.57E+03	6.78E+01	2.89E+03	4.62E+04	5.38E+02	1.07E+03	0.00E+00	0.00E+00	1.02E+03	0.00E+00	0.00E+00	5.42E+02
	**ITLBO**	6.27E+03	5.85E+01	2.56E+03	6.48E+02	2.13E+02	6.75E+02	0.00E+00	0.00E+00	8.86E+02	0.00E+00	0.00E+00	2.65E+02
	**HSTLBO**	6.64E+02	1.88E+01	2.20E+03	9.99E-16	3.92E-15	6.46E+02	3.91E-10	8.16E-11	1.91E+03	0.00E+00	0.00E+00	3.41E+02

### 4.3 Statistical test

To investigate the significant difference between our method and ten compared algorithms, in this section, the Wilcoxon signed rank test is conducted at 5% significance level to judge whether the optimal solutions on 20 independent runs with our method differ significantly from those of compared algorithms. Table 5 records the corresponding p-values for 20 functions between the proposed algorithm and other algorithms, which indicate that all the p-values are less than 0.05. The last three lines of Table 5 records the performance of algorithms, where “+”, “=“, and “-” separately represent the optimal solutions on 20 independent runs of the corresponding algorithm are better than, similar to, and worse than those of HSTLBO. We can see from Table 5 that the performance of proposed algorithm is significantly different with those of other algorithms for each function, and our method is superior to other algorithms for most of functions.

**Table 5:** Results of Wilcoxon’s rank sum test at 0.05 significance level between HSTLBO and other ten algorithms. The p-value is shown (NaN denotes no difference).

Functions	HS	IHS	ITHS	NGHS	EHS	TLBO	ATLBO	WTLBO	TLBO_GC	ITLBO
F1	1.2E-08	1.2E-08	2.9E-04	1.2E-08	1.2E-08	1.0E-09	6.0E-11	6.0E-11	6.0E-11	6.0E-11
F2	1.0E-08	1.0E-08	2.7E-04	1.0E-08	1.0E-08	1.0E-09	1.0E-09	1.0E-09	1.0E-09	1.0E-09
F3	1.2E-08	1.2E-08	1.2E-08	1.2E-08	1.2E-08	1.2E-08	1.2E-08	1.2E-08	1.2E-08	1.2E-08
F4	1.2E-08	1.2E-08	1.2E-08	1.2E-08	1.2E-08	6.0E-11	6.0E-11	6.0E-11	6.0E-11	6.0E-11
F5	1.0E-08	1.0E-08	1.0E-08	1.0E-08	1.0E-08	5.99E-11	5.99E-11	5.99E-11	5.99E-11	5.99E-11
F6	1.2E-08	1.2E-08	1.2E-08	1.2E-08	1.2E-08	1.2E-08	1.2E-08	1.2E-08	1.2E-08	1.2E-08
F7	1.2E-08	1.2E-08	1.2E-08	1.2E-08	1.2E-08	1.2E-09	1.2E-09	1.2E-09	1.2E-09	1.2E-09
F8	1.2E-08	1.2E-08	1.2E-08	1.2E-08	1.2E-08	1.2E-08	1.2E-08	1.2E-08	1.2E-08	1.2E-08
F9	1.2E-08	1.2E-08	1.2E-08	1.2E-08	1.2E-08	1.2E-08	1.2E-08	1.2E-08	1.2E-08	1.2E-08
F10	1.2E-08	1.2E-08	1.2E-08	1.2E-08	1.2E-08	1.2E-08	1.2E-08	1.2E-08	1.2E-08	2.9E-04
F11	1.2E-08	1.2E-08	1.2E-08	1.2E-08	1.2E-08	1.2E-08	1.2E-08	1.2E-08	1.2E-08	1.2E-08
F12	1.2E-08	1.2E-08	1.2E-08	1.2E-08	1.2E-08	1.2E-08	1.2E-08	1.2E-08	1.2E-08	1.2E-08
F13	1.2E-08	1.2E-08	1.2E-08	1.2E-08	1.2E-08	1.2E-08	1.2E-08	1.2E-08	1.2E-08	1.2E-08
F14	1.2E-08	1.2E-08	1.2E-08	1.2E-08	1.2E-08	1.2E-08	1.2E-08	1.2E-08	1.2E-08	1.2E-08
F15	1.2E-08	1.2E-08	1.2E-08	1.2E-08	1.2E-08	1.2E-08	1.2E-08	1.2E-08	1.2E-08	1.2E-08
F16	1.2E-08	1.2E-08	1.2E-08	1.2E-08	1.2E-08	1.2E-08	1.2E-08	1.2E-08	1.2E-08	1.2E-08
F17	1.2E-08	1.2E-08	1.2E-08	1.2E-08	1.2E-08	1.2E-08	1.2E-08	1.2E-08	1.2E-08	1.2E-08
F18	1.2E-08	1.2E-08	1.2E-08	1.2E-08	1.2E-08	1.2E-08	1.2E-08	1.2E-08	1.2E-08	1.2E-08
F19	1.2E-08	1.2E-08	1.2E-08	1.2E-08	1.2E-08	1.2E-09	1.2E-09	1.2E-09	1.2E-09	1.2E-09
F20	1.2E-08	1.2E-08	1.2E-08	1.2E-08	1.2E-08	NaN	NaN	NaN	NaN	NaN
+	0	0	0	0	0	5	5	5	5	5
=	0	0	0	0	0	1	1	1	1	1
-	20	20	20	20	20	14	14	14	14	14

To further detect the significant differences between HSTLBO and ten compared algorithms, the multiple-problem Wilcoxon’s test is employed to check the comparisons. Table 6 records the statistical results, where “W+” is the number of cases in which the null hypothesis was rejected and our method shows a statistically superior performance at the 95% significance level, “W-” denotes the number of cases in which the null hypothesis was rejected and the HSTLBO displays an inferior performance, “W=“ represents the number of cases in which the null hypothesis was accepted [36**] [**37]. We can find from Table 6 that our method has higher “W+” values than “W-” values in all cases, which demonstrates that our method is significantly better than other ten algorithms on 20 test functions.

**Table 6:** Multi-problem based statistical pairwise comparison of HSTLBO and other ten algorithms. (α = 0.05, *D* = 1000).

HSTLBO vs. Algorithm	*D* = 1000				
	P-value	W+	W-	W=	Winner
HSTLBO vs. HS	8.86E-05	210	0	0	HSTLBO
HSTLBO vs. IHS	8.86E-05	210	0	0	HSTLBO
HSTLBO vs. ITHS	8.86E-05	210	0	0	HSTLBO
HSTLBO vs. NGHS	8.86E-05	210	0	0	HSTLBO
HSTLBO vs. EHS	8.86E-05	210	0	0	HSTLBO
HSTLBO vs. TLBO	0.004848	120	70	20	HSTLBO
HSTLBO vs. ATLBO	0.004848	120	70	20	HSTLBO
HSTLBO vs. WTLBO	0.004848	120	70	20	HSTLBO
HSTLBO vs. TLBO_GC	0.004848	120	70	20	HSTLBO
HSTLBO vs. ITLBO	0.00621	116	74	20	HSTLBO

### 4.4 Analysis of exploration and exploitation

In this section, we investigate the convergence of HSTLBO by tracing population diversity in the search process. The population diversity is defined as follows,

$$Diversity=\frac{1}{D}\sum_{i=1}^{D}\sqrt{\frac{1}{NP}\sum_{j=1}^{NP}{{\text{(X}}_{i}^{j}-\;\bar{{\text{X}}_{\text{i}}})}^{2}}$$

**Where** $\bar{{\text{X}}_{\text{i}}}(i=1,2,\ldots ,D)$ denotes the mean value of i^th^ decision variable in population.

Three complex multimodal optimization problems (F_1_:Ackley, F_3_:Levy, F_12_:Griewank Shift) are employed to investigate the balance between the exploration power and the exploitation. During the search process, we trace the changes of population diversity of each compared algorithm.

**Fig 9:**
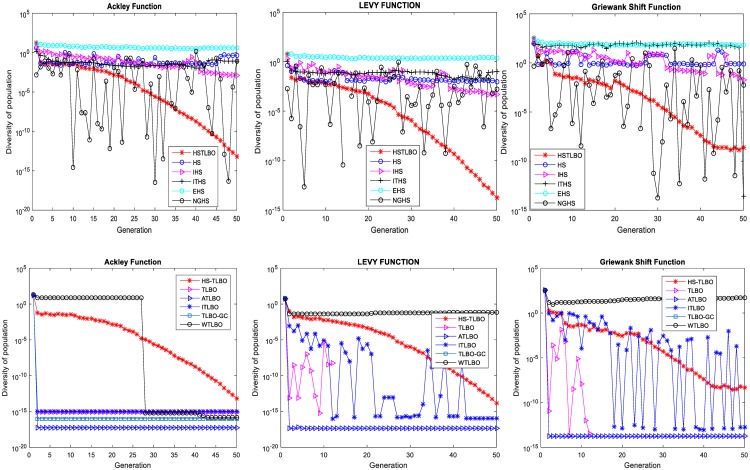
Curves of population diversity of 11 algorithms.

The Fig 9 displays the curve changes of population diversity of eleven algorithms, from which we can easily find that the diversity curves of HSTLBO algorithm decrease gradually during the search process, which are more asymptotic and stable than other algorithms. In this way, the HSTLBO possess strong exploration power in the early stage of search for exploring the unknown search regions, with the search continue, the exploitation power increases and the exploration power decreases gradually, in the later stage, the population has gathered into the globally optimal region. And by this time HSTLBO should have obtained much high exploitation power for exploiting the high precision solution. However, compared with HSTLBO, five HS variations (HS, IHS, ITHS, EHS and NGHS) keep high population diversity from beginning to end, which make them have strong exploration power but exploitation power very weak. Conversely, the diversity of five TLBO variations decrease very quickly in the begin stage, which makes them easily be trapped into local search owing to losing the exploration power prematurely.

## 5. HSTLBO for solving complex portfolio optimization problem

To further investigate the performance of HSTLBO algorithm, complex portfolio optimization problem are employed to test the ability of solving real-world application. The portfolio optimization aims to choose the optimal proportions of various assets for obtaining maximum portfolio return with minimum risk. In this work, we apply HSTLBO algorithm to choose the optimal portfolio proportions for Nikkei 225 stock index that maps companies on the Tokyo Stock Exchange (TSE) (http://en.wikipedia.org/wiki/Nikkei_225) and compare the test results of HSTLBO with four intelligent algorithms (GA, PSO, TS, SA)[38–40]. We employ mean Euclidian distance (MED), variance of returns error (VRE) and mean return error (MRE) as performance indexes that are defined in literatures [39,41].

The test data is from http://people.brunel.ac.uk/~mastjjb/jeb/orlib/portinfo.html and the experiments are performed on two conditions:

1. **Unconstraint**. The portfolio proportion and desired number of investment assets are not constrained.
2. **Constraint.** The portfolio proportion of each asset $x_{i}^{L}=0.0$ and $x_{i}^{U}=1$; the desired number of portfolio selection assets *K* = 10.

In this work, we employ the same method of constraint handle as the literature [41] which can handle the boundary constraint of the portfolio proportion and the desired number of portfolio selection assets very well.

**Fig 10:**
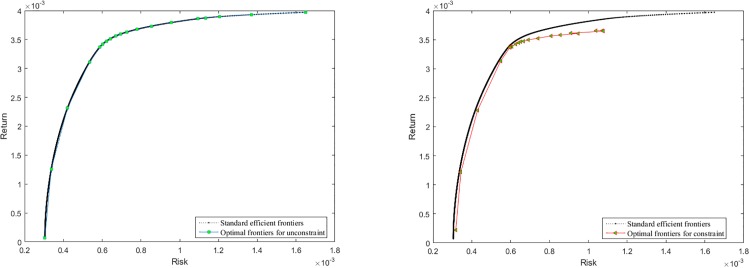
Optimal frontiers for unconstraint and constraint portfolio Nikkei index 225.

The test results on three evaluation indexes (MED, VRE and MRE) are presented in Table 7, and The comparison of efficient frontiers for different constraint conditions are shown in Fig 10. We can see easily from Table 7 that our method obtains more outstanding performance on MED, VRE and MRE than modified GA, PSO, TS and SA. From Fig 10, we can find that optimal frontiers of our method are almost overlapped with standard efficient frontiers for unconstraint CCMV model, and for constraint CCMV model, the optimal frontiers of our algorithm are also very close to the standard efficient frontiers that are obtained without considering the constraint conditions. Therefore, our approach is effective for solving complex portfolio optimization problems.

**Table 7:** Simulation results of five algorithms on Nikkei index 225.

	Unconstraint					Constraint				
	GA	PSO	TS	SA	HSTLBO	GA	PSO	TS	SA	HSTLBO
MED	1.50E-03	2.90E-04	1.50E-04	1.90E-04	8.33E-07	9.93E-03	1.90E-03	1.00E-03	1.23E-03	6.63E-05
VRE	2.10E-01	4.30E-01	2.20E-01	2.10E-01	6.36E-02	1.21E+00	2.43E+00	1.24E+00	1.20E+00	5.23E+00
MRE	9.30E-01	1.40E-01	7.40E-02	7.20E-02	1.34E-02	5.33E+00	8.00E-01	4.21E-01	4.13E-01	1.35E+00

## 6. Conclusion

Both Harmony Search and Teaching-Learning-Based Optimization are new swarm intelligent optimization algorithms, which have got much attention in recent years. In this work, in order to improve the performance of HS and TLBO, a hybrid HSTLBO algorithm is presented.

In the HSTLBO, both HS and TLBO are improved to enhance the global search ability. A self-adaptive selection strategy is presented to balance the exploration power and the exploitation power. At the early stage of search process, the HS algorithm gets a higher opportunity than TLBO, which aims to explore the unknown regions and avoid lose the globally optimal solution. With the increasing number of iteration, the opportunity of TLBO is raised step by step. At the later stage of search, when the population has gathered into one region which maybe contains the global optimal solution, the TLBO has obtained much more opportunity for exploiting high precision optimal solution.

The experimental results also demonstrate that our method is a promising optimization algorithm in solving large scale and complex optimization problems.
